# Frequency of child maltreatment in a representative sample of the German population

**DOI:** 10.1186/1471-2458-13-980

**Published:** 2013-10-20

**Authors:** Benjamin Iffland, Elmar Brähler, Frank Neuner, Winfried Häuser, Heide Glaesmer

**Affiliations:** 1 Department of Psychology, Bielefeld University, Postbox 100131, 33501 Bielefeld, Germany; 2 Department of Medical Psychology and Medical Sociology, University of Leipzig, Leipzig, Germany; 3 Department of Internal Medicine, Klinikum Saarbrücken, Saarbrücken, Germany; 4 Department of Psychosomatic Medicine, Technische Universität München, Munich, Germany

**Keywords:** Child maltreatment, Childhood abuse and childhood neglect

## Abstract

**Background:**

Representative data about the frequency of child maltreatment is needed in order to estimate the extent of the problem in the wider population as well as to provide the basis for interpretation of frequency rates in clinical samples. However, previous representative studies on the frequency of child maltreatment in Germany and other countries were limited as they focused on the assessment of physical and sexual abuse whilst emotional forms of maltreatment were ignored. In addition, previous studies applied scales that had not been validated against external criteria.

**Methods:**

In a cross-sectional study, standardized questionnaires were administered to a representative sample of the German population. Maltreatment in childhood and adolescence was assessed using the German version of the Childhood Trauma Questionnaire. Empirically derived threshold values for the five different types of child maltreatment including emotional maltreatment were applied to determine presence of abuse and neglect.

**Results:**

Complete data was available from *N* = 2,500 subjects. Prevalence rates were 13.9% for emotional neglect, 10.2% for emotional abuse, 12.0% for physical abuse, 48.4% for physical neglect, and 6.2% for sexual abuse. Differences between sexes were found for the frequency of sexual abuse.

**Conclusions:**

Although our analysis has found lower rates of child maltreatment than previous reports that used less well validated criteria, the results of this study confirm that child abuse, with its many different facets, is a significant problem in Germany.

## Background

Child maltreatment refers to all forms of abusive and neglectful behavior involving emotional, physical and sexual transgressions, resulting in actual or potential harm to the child’s health, survival or development [1,2]. Although child maltreatment is one of the most important social challenges worldwide and is associated with substantial impairments of social wellbeing and health [3-6], information about the frequency of child maltreatment in high-income societies is still scarce and inconsistent.

For example, there are hardly any studies that have reported rates of child maltreatment in the German population. As an exception Wetzels [7] has included an instrument for the assessment of sexual and physical abuse in a representative survey of 3,289 subjects in the age range of 16 to 59. Sexual abuse was reported by 8.6% of the women and 2.8% of the men and prevalence rate of physical abuse was 10.6%. However, this study is limited by the fact that the assessment of child maltreatment was not comprehensive. Although consensus definitions of child maltreatment include five types, i.e. physical abuse, physical neglect, emotional abuse, emotional neglect and sexual abuse [8-10], and the emotional types of maltreatment seem to have similar detrimental health effects as sexual and physical abuse [11], the emotional forms of maltreatment have commonly been excluded from large-scale representative surveys in different countries [12-15]. In addition, similar to the other studies Wetzels [7] concentrated on the assessment of physical and sexual abuse using an *ad-hoc* developed scale that does not allow a comparison of results with other surveys or data from clinical populations that used different questionnaires.

Recently, Haeuser et al. [16] reported the frequency of child maltreatment from a representative survey in Germany. This study is outstanding since data on child maltreatment was collected using the standard and well validated child maltreatment instrument Childhood Trauma Questionnaire (CTQ) [17] that allows the assessment of all common types of child maltreatment. The strength of this instrument is that it allows a severity rating of sexual abuse, physical abuse, physical neglect, emotional abuse and emotional neglect, which reflects the fact that the different forms of child maltreatment may be continuous phenomena ranging from small to severe transgressions rather than clearly delimitable entities [18,19]. However, a dichotomous categorization of the forms of child maltreatment using a severity cut-off is indicated to allow the comparison of rates of child maltreatment from studies that applied different instruments based on similar definition. While the authors of the CTQ do not provide thresholds to determine the presence of the different types of maltreatment on a dichotomous basis, Haeuser et al. [16] reported frequency rates based on severity ratings above the severity range labeled as 'low to moderate’. The resulting prevalence rates were as high as for emotional (49.5%) and physical neglect (48.4%), with 12% for physical abuse, 15% for emotional abuse and 12,6% for sexual abuse.

The present article presents a re-analysis of the German general survey data which had also been used by the Haeuser et al. [16] study. In contrast to the previous analysis, this analysis did not rely on cut-off scores based on the original severity ratings, but on empirically determined and validated threshold values for the different types of child maltreatment as reported by Walker et al. [20]. These cut-off criteria had been ascertained by relating CTQ subscale scores to ratings of experts blind for the CTQ scores who administered detailed clinical interviews. Based on the fulfillment of consensus child abuse and neglect criteria [20], experts determined whether participants had a history of clinically significant abuse or neglect. Walker et al. [20] used the same definitions of child abuse and neglect the items of the five subscales of the CTQ were derived from. Emotional abuse was defined as “verbal assaults on a child’s sense of worth or well-being or any humiliating or demeaning behavior directed toward a child by an adult or older person”. Emotional neglect was defined as “the failure of caretakers to meet children’s basic emotional and psychological needs, including love, belonging, nurturance, and support”. Physical abuse was defined as “bodily assaults on a child by an adult or older person that posed a risk of, or resulted in, injury”. Physical neglect was defined as “the failure of caretakers to provide for a child’s basic physical needs, including food, shelter, clothing, safety, and health care” (poor parental supervision was also included if it placed a child’s safety in jeopardy). Sexual abuse was defined as “sexual contact or conduct between a child younger than 17 years of age and an adult or older person (at least 5 years older than the child)”. Receiver operating characteristic (ROC) methods had been employed to determine threshold scores for each subscale. Resulting threshold scores showed good to excellent sensitivity and specificity. Maltreatment is assumed when threshold scores for emotional abuse (10), emotional neglect (15), physical abuse (8), physical neglect (8), and sexual abuse (8) are met. In contrast threshold scores that were established by Bernstein et al. [18] and used in the Haeuser et al. [16] study were 9 for emotional abuse, 10 for emotional neglect, 8 for physical abuse, 8 for physical neglect, and 6 for sexual abuse. With this procedure we aim to provide the first comprehensive and representative prevalence data on different types of child maltreatment based on empirically derived cut-off criteria for the German population. Empirically derived and externally validated cut-off criteria allow for a more accurate and clinically significant evaluation of the presence of a history of abuse and neglect while clinical relevance of the cut-off scores used in previous studies remain uncertain. In addition, inter-correlations and co-occurrence of different kinds of maltreatment as well as their association to age and sex were examined.

## Methods

### Subjects

A representative sample of the German general population was selected with the assistance of a demographic consulting company (USUMA, Berlin, Germany). The area of Germany was separated into 258 sample areas representing the different regions of the country. Households of the respective area and one member of this household fulfilling the inclusion criteria (age at or above 14, able to read and understand the German language) were selected randomly through the Kish-selection-grid technique. The Kish-selection-grid technique aims to sample individuals on the doorstep. The system is devised so that all individuals in a household have an equal chance of selection. The sample was designed to be representative in terms of age, gender, and education. A first attempt was made for 4,455 persons. If not at home, a maximum of three attempts were made to contact the selected person. All subjects were visited by a study assistant, informed about the investigation (covering several research questions), and self-rating questionnaires were presented. All participants signed an informed consent. When under the age of 18, the parents were asked to sign a written consent. The assistant waited until participants answered all questionnaires and offered help if persons did not understand the meaning of questions. A total of 2,500 people between the ages of 14 and 90 years agreed to participate and completed a questionnaire on physical and mental health which included socio-demographic information as well as the German version of the CTQ. Data was collected in April 2010. The response rate reached 56%. All relevant international guidelines and ethical standards relating to the collection of personal data from human beings have been abided. Participants were recruited by a professional demographic consulting company (USUMA, Berlin, Germany) that abides to the ICC/ESOMAR International Code on Market and Social Research regarding ethics in social sciences research [21]. This code embodies the highest professional and ethical standards relating to market and social research, and guarantees informed consent and the anonymous processing of personal data. Additionally, the company conducting data sampling is a member of a group with general ethical approval from the German government to conduct these types of surveys. According to the Federal Data Protection Act (Bundesdatenschutzgesetz BDSG, § 30a), the need for consent from a specific ethics commitee is waived for USUMA surveys.

The German version of the CTQ [22] is a 28-item self-rating scale consisting of five subscales. The items are rated from 1 (never true) to 5 (very often true) with a possible range of subscale scores of 5 to 25. The German version of the CTQ has been shown to be a reliable and valid screening tool for childhood maltreatment in clinical samples [22,23]. However, in a recent validation study from the general population [24], the five factor structure of the original version showed only a moderate fit. The subscale 'physical neglect’ was highly correlated with the other subscales and presented a weak internal consistency in comparison to other subscales. The fit of a four factor structure excluding the physical neglect items was superior to the five factor model, suggesting that the CTQ would benefit from disregarding the physical neglect subscale [24]. Nevertheless, prevalence rates for the physical neglect subscale will be presented here, since it is widely used in clinical research.

In the present study, threshold scores suggested by Walker et al. [20] were applied to determine presence of the different types of child maltreatment. In addition, three of the items of the CTQ are used as a minimization/denial scale to identify cases with problematic validity. In this scale, only extreme scorings (score 5) are counted. Three such extreme scorings should be regarded as an indication of probable minimization, unrealistic statements and severe psychological defences [17]. In the following analyses, participants were categorized as minimizing if they score 'very often true’ on all of the three items of this scale.

### Statistical analyses

The population was divided into groups according to fulfillment of the different types of maltreatment. For each type of maltreatment, these groups were compared pair-wise using the Student *t* test for independent groups for continuous normally distributed dependent variables. The Mann–Whitney *U* test was used for ordinally scaled data or data from skewed distributions. Statistical associations among categorical variables were analyzed using Pearson’s chi-square test and the Fisher exact test. Associations among ordinally scaled variables were estimated using the Spearman rank order correlation.

## Results

### Demographics

The CTQ and the other baseline assessment instruments were fully completed by 2,500 subjects. The average age of the respondents was *M* = 50.66 (*SD* = 18.56). Table 1 presents participants’ demographics and means on the assessments.

**Table 1:** **Subject characteristics and mean values on psychopathology (*N*** **= 2,500)**

Age, *M* (*SD, range*)	50.66 (18.56, 14–90)
Gender, % male (*n*)	46.90 (1172)
Family status, % single (*n*)	39.40 (986)
Childhood Trauma Questionnaire, *M* (*SD*)	35.99 (10.48)^a^
Emotional Abuse, *M* (*SD*)	6.51 (2.60)
Emotional Neglect, *M* (*SD*)	10.09 (4.23)
Physical Abuse, *M* (*SD*)	5.88 (2.17)
Physical Neglect, *M* (*SD*)	8.15 (3.02)
Sexual Abuse, *M* (*SD*)	5.45 (1.66)
Minimization/Denial, *M* (*SD*)	10.73 (2.78)

Note: ^a^CTQ sum score for the 28-item version.

We found that 13.9% of the subjects went beyond the pre-established threshold for emotional neglect, 10.2% for emotional abuse, 12.0% for physical abuse, 48.4% for physical neglect, and 6.2% for sexual abuse (Table 2). Sexual abuse was significantly more prevalent among women. No differences between sexes were found for the other types of abuse (Table 2). Cumulative frequencies of the different types of child maltreatment are presented in Table 3. In our sample, 33.9% reported experience of at least one type of abuse. All subtypes of maltreatment were significantly intercorrelated. Emotional and physical abuse showed a moderate correlation, whereas the other correlations were rather small (Table 4).

**Table 2:** **Prevalence of child abuse by trauma type and comparison of childhood abuse type by mean age (*N*** **= 2,500)**

	**Sex**							**Age**				
	**Total**		**Male**		**Female**			**Abused**		**Non-abused**		
	***n***	***%***	***n***	***%***	***n***	***%***	***p***	**Mean age**	***SD***	**Mean age**	***SD***	***p***
Emotional Abuse	254	10.2	109	9.3	145	10.9	.184^a^	50.60	18.65	50.67	18.56	.954^b^
Emotional Neglect	348	13.9	171	14.6	177	13.4	.374^a^	51.50	17.46	50.51	18.72	.444^c^
Physical Abuse	301	12.0	149	12.7	152	11.5	.335^a^	54.09	18.88	50.18	18.48	.001^b^
Physical Neglect	1210	48.4	580	49.5	630	47.5	.305^a^	55.76	18.21	45.81	17.54	.000^c^
Sexual Abuse	156	6.2	44	3.8	112	8.4	.000^a^	52.03	17.68	50.56	18.62	.339^b^
								**Minimizing**		**Non-minimizing**		
Minimization/Denial	214	8.6	94	8.0	120	9.0	.365^a^	44.27	18.34	51.25	18.47	.000^b^

Test statistic: ^a^Pearson chi square, ^b^two sample *t* test, ^c^Mann–Whitney *U* test.

**Table 3:** Frequency of total number of abuse types

**Numbers of types of abuse reported**	***n***	***%***
0	1133	45.4
1	847	33.9
2	295	11.8
3	94	3.8
4	82	3.3
5	42	1.7

Test statistic: Pearson chi square, *p* < .001.

**Table 4:** Correlation coefficients of abuse types

	**Emotional abuse**	**Emotional neglect**	**Physical abuse**	**Physical neglect**	**Sexual abuse**
Emotional Abuse	1.00				
Emotional Neglect	.364	1.00			
Physical Abuse	.461	.293	1.00		
Physical Neglect	.218	.328	.232	1.00	
Sexual Abuse	.353	.196	.338	.154	1.00

Spearman rank order correlation matrix. All correlations were significant, *p* < .001.

For each trauma type, mean ages of subjects meeting the threshold were compared to the mean ages of those who did not. Significant differences were found for physical abuse and physical neglect. Respectively, subjects who met the thresholds were significantly older than subjects who did not (Table 2).

On the minimization/denial scale, 8.6% of the participants endorsed all items. No sex differences could be found on this scale, while participants who were minimizing were significantly younger than participants who did not minimize (Table 2).

## Discussion

In this study, we presented prevalence rates of child abuse in a representative sample of the German population based on empirically derived cut-off scores. The frequency of emotional neglect was 13.9%, 10.2% of the subjects reported emotional abuse, 12.0% met criteria for physical abuse, 48.4% for physical neglect, and 6.2% for sexual abuse.

Unsurprisingly, as all of the cut-off values used in our analysis were lower than those applied in the previous analysis of this dataset reported by Haeuser et al. [16], frequencies reported here were lower on all subscales except for the physical abuse and the physical neglect scale (a survey of prevalence rates presented in previous studies is given in Table 5). In particular, our cut-off resulted in considerably lower frequencies for emotional neglect. Previous representative surveys in Germany [7,25,26] were restricted to the assessment of physical or sexual abuse. While the magnitudes of physical abuse in the studies by Wetzels [7] as well as Glaesmer et al. [25] were tentatively comparable, Hauffa et al. [26] reported an exceptionally low rate of physical violence. Since regional or temporal factors as well as the use of different assessments are not sufficient to explain these differences, the authors suggested that the answering patterns of subjects may account for the discrepancy in reported frequencies [26].

**Table 5:** Survey of prevalence rates reported in previous studies (in %)

	**Emotional abuse**	**Emotional neglect**	**Physical abuse**	**Physical neglect**	**Sexual abuse**
Present Study	10.2	13.9	12.0	48.4	6.2
Glaesmer et al. [25]	-	-	8.5	-	1.0
Green et al. [27]	-	-	8.4	5.6^a^	6.0
Haeuser et al. [16]	15.0	49.5	12.0	48.4	12.6
Hauffa et al. [26]	-	-	3.9	-	1.2
Thombs et al. [28]	30.6	-	16.5	-	10.3
von Sydow [29]	-	-	-	-	18-21
Wetzels [7]	-	-	10.6	-	5.7

Note: ^a^In this study, there was no distinction made between emotional and physical neglect. Neglect presented here was the frequency of not having adequate food, clothing, or medical care, having inadequate supervision, and having to do age-inappropriate chores.

Frequencies of sexual abuse reported in previous representative surveys showed a wide range. While Wetzels [7] reported a rate that was comparable to our findings, frequencies of sexual abuse in the studies of Glaesmer et al. [25] and Hauffa et al. [26] were rather small. Comparison of prevalence rates for sexual abuse is limited by the fact that Glaesmer et al. [25] as well as Hauffa et al. [26] reported frequencies of subjects being raped. Criteria for sexual abuse used in our study embodied a wide range of sexual assaults including rape. The wide range of criteria for sexual abuse may account for higher prevalence rates for both sexes in our study compared to previous findings.

In a survey of 91 women born in the years 1895 to 1936, von Sydow [29] reported much higher prevalence rates for sexual abuse. Experiences of sexual abuse under the age of 12 were reported by 18% of the women, 21% reported sexual abuse between the age of 13 and 21.

A recent validation study of the German translation of the CTQ has indicated that the scale for physical neglect has weak psychometric properties, is highly correlated with the other subscales and presented with a weak internal consistency in comparison to the other subscales [24]. These factors may have contributed to the uncommon and possibly excessive rates of physical neglect found in this study. As a consequence, findings based on this subscale from our study as well as from other studies should be interpreted with caution.

In our study, sex differences in the frequencies of child abuse were only found for sexual abuse. Prevalence rates of physical and sexual abuse reported for both sexes were comparable to frequencies reported by Wetzels [7]. In their sample, 11.8% of the men and 9.9% of the women experienced a history of physical maltreatment in childhood. A history of sexual abuse was reported for 2.8% of the men and 8.6% of the women. In contrast to the findings of Glaesmer et al. [25], prevalence rates of physical abuse in women were higher. While Glaesmer et al. [25] reported a frequency of 5.1%, 11.5% of the women in our sample met criteria for physical abuse.

In a population-based study in the US, the CTQ short form was administered in a randomized telephone interview survey with adults aged 18 to 65 [28]. Respondents were classified as having been abused if they either explicitly labeled themselves as having been abused or rated anything other than “never” to the single item of the various CTQ subscales that explicitly used the term 'abused’. Prevalence rates for the subscales physical, emotional and sexual abuse were presented. Findings from our German sample were lower on all subscales. However, these differences might be attributed to differences in sampling as well as in the applied cut-off values. It is noteworthy that, despite the differences in measurement, the replication of the National Comorbidity Survey (NCS-R) presented prevalence rates for child adversities similar to the rates reported in our study [27].

The intercorrelations and co-occurrence of maltreatment types presented in this study were consistent with previous reports [7,16,20,30]. All types of maltreatment were significantly inter-correlated. In line with findings of Haeuser et al. [16], the smallest relationship was found for emotional neglect and sexual abuse. In our study, fulfillment of multiple types of maltreatment was reported for 20.6%. In the sample of Walker et al. [20] 23% of the subjects met criteria for more than one type of abuse. Reports of Haeuser et al. [16] were even higher. In their study, 40.3% of the participants reported at least two kinds of maltreatment. When the physical neglect subscale is excluded, in our study merely 9.6% of the subjects fulfill criteria for multiple types of maltreatment. This rather small frequency suggests that high frequencies of co-occurrence in prior studies result from the inclusion of the CTQ physical neglect subscale and the use of cut-off values that were based on severity ratings.

Limitations of our study include the fact that the assessment of childhood maltreatment is based on retrospective accounts and self-report, which is subject to recall biases [16]. However, the analysis of that recall bias indicates that these distortions are not sufficiently large to invalidate retrospective reports in general [31]. In addition, the present study is limited by a response rate of 56%. Prior surveys conducting a similar technique to recruit subjects reached higher response rates (62.1%) [32]. Due to data protection, differences between responders and non-responders on clinical and socio-demographical data could not be analyzed. Therefore, frequencies may have been affected by participation bias. Particularly, assessment of trauma associated experiences might have caused avoidance and refusal to participate.

## Conclusion

In summary, findings from our study confirmed that methodological details, in particular the definition of maltreatment types and cut-off values, have a substantial impact on the frequency rates determined in surveys. However, a validation of the cut-off scores used in the present study in a German sample would be desirable in order to achieve more accurate assessments of prevalence rates. Furthermore, the present study is to our knowledge the first to present prevalence rates for the minimization/denial scale in a representative sample. In general, our analysis based on empirically derived cut-off values resulted in lower rates of child maltreatment than in previous reports that used less well validated criteria. However, still as many as 33.9% (Table 3) reported experience of a type of maltreatment, which demonstrates that child maltreatment is a significant social problem in Germany like in other countries world-wide. Commonly, prevention efforts are restricted to targeting the reduction of sexual or physical abuse. Our findings show that emotional types of abuse and neglect are at least as common and that new and more comprehensive forms of child protection may be indicated. Prevalence rates reported in our study allow for more thorough evaluation of data found for child maltreatment in clinical samples and in future research.

## Competing interests

The authors declare that they have no competing interests.

## Authors’ contributions

BI performed the statistical analyses and interpretation of findings, and drafted the manuscript. EB participated in the conception and design of the study, and data collection. FN made substantial contributions to the statistical analyses and interpretation of findings, helped to draft and revised the manuscript. WH participated in the conception and design of the study, and data collection. HG participated in the conception and design of the study, collected data, made substantial contributions to the statistical analyses and interpretation of findings, and revised the manuscript. All authors read and approved the final manuscript.

## Pre-publication history

The pre-publication history for this paper can be accessed here:

http://www.biomedcentral.com/1471-2458/13/980/prepub
